# Prenatal substance use during the COVID-19 pandemic in the United Kingdom: associations with depression, anxiety, and pandemic stressors

**DOI:** 10.3389/fpubh.2026.1760266

**Published:** 2026-03-19

**Authors:** Swarali Datye, Eva M. J. Peters, Anita C. Windhorst, Edwin van Teijlingen, Alison MacRae-Miller, Latha Vinayakarao, Minesh Khashu, Fabian B. Fahlbusch, Melanie L. Conrad

**Affiliations:** 1 Section of Neonatology and Pediatric Intensive Care, Department of Pediatrics and Adolescent Medicine, Faculty of Medicine, University of Augsburg, Augsburg, Germany; 2 Institute of Microbiology, Infectious Diseases and Immunology, Berlin Institute of Health, Charité Universitätsmedizin Berlin, Corporate Member of Freie Universität Berlin, Humboldt-Universität zu Berlin, Berlin, Germany; 3 Psychoneuroimmunology Laboratory, Department of Psychosomatic Medicine and Psychotherapy, Justus-Liebig University, Giessen, Germany; 4 Department of Psychosomatic Medicine and Psychotherapy, Charité Universitätsmedizin Berlin, Berlin, Germany; 5 Institute of Medical Informatics, Justus-Liebig-University Giessen, Giessen, Germany; 6 Center for Midwifery and Women's Health, Bournemouth University, Bournemouth, United Kingdom; 7 Department of Family Medicine, University of British Columbia, Vancouver, BC, Canada; 8 University Hospitals Dorset NHS Foundation Trust, Poole, United Kingdom; 9 Institute of Medical Psychology, Berlin Institute of Health, Charité Universitätsmedizin Berlin, Corporate Member of Freie Universität Berlin, Humboldt-Universität zu Berlin, Berlin, Germany

**Keywords:** alcohol, co-use, COVID-19 pandemic, depression, prenatal substance use, tobacco, United Kingdom

## Abstract

**Introduction:**

The COVID-19 pandemic was associated with high levels of depression and anxiety among pregnant individuals in the UK, as shown previously in the EPPOCH cohort. However, the relationships between these psychological burdens, pandemic-related stressors, and substance use in the prenatal period have not been systematically investigated.

**Methods:**

To address this gap, we conducted a cross-sectional analysis of baseline EPPOCH data (*n* = 3,292; June–November 2020). Participants reported alcohol, tobacco, cannabis, and illicit drug use before and after recognition of pregnancy, alongside validated measures of depression, anxiety, pregnancy-related anxiety, and pandemic stressors. Linear regression models examined associations between mental health, COVID-19 stressors, and substance use after pregnancy recognition. A qualitative thematic analysis of 380 open-ended responses explored perceptions of substance use post-pregnancy recognition.

**Results:**

Alcohol was the most commonly used substance before pregnancy. Following pregnancy recognition, tobacco (8.75%), and alcohol (8.60%) were the most frequently reported substances, followed by cannabis (1.49%) and illicit drugs (0.12%). Tobacco use after pregnancy recognition was associated with higher levels of depressive symptoms and pandemic stressors, including perceived personal health threat and not receiving necessary care. Prenatal co-use of substances was associated with higher depressive symptoms and pandemic-related financial difficulties. Qualitative themes included continued substance use until pregnancy detection, vaping as a perceived safer-use strategy, and midwifery advice influencing prenatal substance use decisions.

**Discussion:**

In this large UK pregnancy cohort recruited during the COVID-19 pandemic, substance use following pregnancy recognition-particularly tobacco-was linked to depression and pandemic-related stressors. These findings highlight the importance of equipping midwives and other healthcare professionals with clear, evidence-based guidance on prenatal substance use, particularly during global health crises.

## 1 Introduction

Maternal health behaviors during pregnancy critically shape offspring health, with consequences extending from perinatal outcomes to long-term development (1). Among these, prenatal alcohol, tobacco, and cannabis use remain major public health concerns with well-established risks for maternal and fetal health (2). Alcohol is particularly harmful because of its “all-or-nothing” impact in early embryogenesis: even if discontinued once pregnancy is recognized, exposure before that point can still result in fetal alcohol spectrum disorders (FASD), which encompass growth restriction, congenital anomalies, cognitive impairment, and behavioral difficulties (3–5). Prenatal tobacco use exposes the fetus to nicotine, carbon monoxide, and other toxins that impair oxygen delivery and increase risks of preterm birth and low birth weight (6, 7). In addition, infants exposed to tobacco in utero may exhibit withdrawal-like symptoms after birth, including irritability, tremors, and feeding difficulties (8, 9). Prenatal cannabis use has been associated with impaired placental function, altered fetal brain development, and adverse neurodevelopmental outcomes (10–13). The use of other illicit substances, including opioids and cocaine, further compounds risks, such as neonatal abstinence syndrome and stillbirth (14, 15).

Beyond their direct biological effects, prenatal substance use is also shaped by psychosocial context. Psychological distress is a well-established driver of substance use, with alcohol, tobacco, and cannabis often adopted as maladaptive coping strategies (16). Pregnancy represents a particularly vulnerable period, when stress can both exacerbate mental health problems and increase substance use. The COVID-19 pandemic introduced additional stressors, including financial instability, social isolation, and disrupted healthcare access that disproportionately affected pregnant individuals (17–19). In the EPPOCH cohort, we previously reported higher depression rates among pregnant individuals in the United Kingdom (UK) compared with five other high-income countries (20). These findings are particularly concerning in the context of the COVID-19 pandemic, given the UK's high prevalence of alcohol use in pregnancy (41–75% depending on measurement and timing) and one of the highest reported FASD rates worldwide (3.2%) (2, 21, 22). Policymakers in the UK have repeatedly highlighted the pressing need to reduce prenatal alcohol exposure (23).

Despite the well-established risks of prenatal substance use and the evidence of deteriorating maternal mental health in the UK during the pandemic, how these domains intersect remains poorly understood. To our knowledge, no UK-based investigation has examined how depression, anxiety, and COVID-19 related stressors intersect with substance use into pregnancy. This study addresses this gap by analyzing data from a large UK pregnancy cohort recruited during the COVID-19 pandemic (EPPOCH; *n* = 3,292). We examined prenatal alcohol, tobacco, and cannabis use in relation to depression, anxiety, and pandemic-related stressors. In addition, qualitative analysis of participants' self-reported perceptions provided further insight into decision-making regarding prenatal substance use. These findings aim to inform evidence-based counseling and public health strategies to mitigate substance-related risks during pregnancy, particularly in times of global health crises.

## 2 Methods

### 2.1 Study design and participants

This study was a cross-sectional analysis of baseline data from the EPPOCH study (Effect of the Pandemic on Pregnancy Outcomes and Childhood Health), a population-based cohort of pregnant individuals recruited across the UK between June and November 2020. Recruitment procedures, eligibility criteria, and cohort characteristics are described in detail elsewhere (20). Briefly, participants were eligible if they were in any stage of a confirmed pregnancy, residing in the UK, and able to complete questionnaires in English. Recruitment occurred primarily via social media platforms that directed participants to the study website, and all data were collected online through REDCap, a secure, web-based platform (24, 25). At enrollment, pregnant individuals completed questionnaires including obstetric information, mental health, pandemic stress and substance use during the COVID-19 pandemic. Ethical approval was obtained from Bournemouth University (UK) and Charité Universitätsmedizin Berlin (Germany). Reporting follows the STROBE guidelines for observational studies.

### 2.2 Measures

#### 2.2.1 Demographic and obstetric characteristics

Participants reported age, education, household income, ethnicity, country of birth, and marital/partnership status. Obstetric data included gestational age at enrollment (calculated from the due date and the date of survey completion), number of previous pregnancies, history of miscarriage(s), and number of live-born children.

#### 2.2.2 Substance use

Participants reported alcohol, tobacco, cannabis, and illicit drug use in the year prior to pregnancy as well as after recognition of pregnancy. For each substance, respondents indicated frequency (days per week) and average quantity consumed per day. Alcohol intake was recorded as number of standard drinks, aided by a visual reference. Tobacco and cannabis use were recorded as number of products per day. Illicit drugs were defined in the questionnaire as illegal substances (e.g., cocaine, methamphetamines, opioids) and did not include non-medical use of prescribed medications. In this study, the term “*prenatal substance use*” was defined as any self-reported use following pregnancy recognition. Co-use was defined as concurrent use of two or more of alcohol, tobacco, or cannabis after recognition of pregnancy. Given the very low prevalence of illicit drug use after pregnancy recognition (0.12%), this variable was not included in correlation or regression analyses.

#### 2.2.3 Mental health

Depressive symptoms were assessed using the Edinburgh Postnatal Depression Scale (EPDS; range 0–30), with scores ≥13 indicating clinically significant symptoms (26). Anxiety symptoms were measured using the PROMIS Anxiety Adult 7-item short form (T-score range 36.3–82.7); scores ≥60 denote moderate-to-severe anxiety (27). Pregnancy-related anxiety was assessed with the 10-item Pregnancy-Related Anxiety Questionnaire (PRAQ; range 10–40), for which no validated cut-off exists (28). For descriptive statistics, measures with validated cut-offs (EPDS, PROMIS) were reported both continuously and categorically, whereas measures without validated cut-offs (PRAQ) were reported only as continuous scores. All regression analyses used continuous scores. Additionally, pre-existing mental health diagnoses were self-reported by participants but not verified against medical records.

#### 2.2.4 COVID-19 stressors

Pandemic stress associated domains were adapted from our sister study, called Pregnancy During the Pandemic (PdP) in Canada (29). Items included financial difficulties, perceived adequacy of maternal/fetal healthcare, perceived threat to self or baby and loneliness experienced during the pandemic. Items were rated on 0–100 visual analog scales, with higher scores indicating greater perceived stress. The impact of the pandemic on social relationships was measured by asking, “How has the COVID-19 pandemic affected your relationship with your circle of friends and family outside of your household?” on a scale of 0–100 (0 = It has strained our relationship, 100 = It has brought us closer together). Lastly, psychological distress at the pandemic peak and in the week prior to recruitment was rated on a 10-point scale on the Distress Thermometer (DT) (0 = no distress, 10 = extreme distress) (30).

### 2.3 Data analysis

#### 2.3.1 Statistical analysis

The 3,403 records collected were manually reviewed and records identified as duplicates or involving participants not pregnant at enrollment were excluded. Missing data were handled using listwise deletion. Descriptive statistics summarized cohort characteristics and substance use prevalence. Paired changes in substance use indices (alcohol, tobacco, cannabis, and illicit drugs) before and after pregnancy recognition were examined using Wilcoxon signed-rank tests. Wilcoxon effect sizes were calculated as *r* = |z|/√N, where *N* represents the number of paired observations included in each test. All tests were two-sided. Spearman's bivariate correlations were used to assess the strength and direction of associations between continuous measures of prenatal substance use, maternal mental health, and COVID-19-related stressors, given the non-normal distributions of these variables. These correlation analyses were conducted to provide descriptive context, and unadjusted *p*-values are reported.

Linear regression models examined four domains: (1) mental health and prenatal substance use; (2) COVID-19 stressors and prenatal substance use; (3) mental health and prenatal substance co-use; and (4) COVID-19 stressors and prenatal substance co-use. Depression (EPDS) and anxiety (PROMIS) were included as representative mental health variables. All models were adjusted for relevant covariates such as demographics (age, education, income), substance use prior to pregnancy, mental health before and during pregnancy and COVID-19 stressors. For regression analyses, false discovery rate was controlled using the Benjamini-Hochberg (BH) procedure, whereby statistical significance was determined by comparing observed *p*-values to BH-adjusted significance thresholds. Analyses were performed using IBM SPSS Statistics version 27 (IBM Corp.).

#### 2.3.2 Qualitative analysis

A total of 380 participants provided free-text responses to the open-ended item: “If you would like to tell us anything else about your use of alcohol, tobacco, cannabis, or illicit drugs while pregnant, please do so here.” Responses were analyzed in NVivo (QSR International) following Braun and Clarke's approach to thematic analysis (31). Two researchers independently coded responses, iteratively refining themes, and representative quotations were selected to illustrate key themes.

## 3 Results

Of 3,403 records collected, 3,292 eligible cases were included in the analysis after removal of 111 duplicates and invalid entries.

### 3.1 Patient demographics

The mean maternal age at enrollment was 31.5 years (SD 5.19; range 19–47), and the mean gestational age at enrollment was 24.7 weeks (SD 9.26). At the time of survey, 48.06% participants were primigravida and 51.64% were multigravida. The cohort was predominantly Caucasian (95.81%) and UK-born (91.28%). Regarding socioeconomic indicators, 36.63% held a bachelor's degree and just under 44% reported an annual household income below £40,000 in 2019 (Table 1).

**Table 1:** Descriptive statistics: demographics, substance use, mental health, and COVID-stressors in the EPPOCH cohort at enrollment.

**Participant characteristics**	***N***	**Prevalence in %**	**Mean**	**SD**	**Median**	**Min**	**Max**
**Basic sociodemographics**							
Age (years)	3,291		31.50	5.19	32	19	47
Education	3,292						
1. Less than high school diploma		1.34					
2. Completed high school		14.09					
3. Completed trade, technical, vocational school or business/community college		29.59					
5. Bachelor's degree		36.63					
5. Graduate degrees (Master, PhD, MD, JD, DDS)		18.35					
Household income (2019)	3,292						
1. Less than £20,000		16.10					
2. £20,000–£39,999		27.73					
3. £40,000–£69,999		38.03					
4. £70.000–£99,999		13.61					
5. £100,000–£200,000		4.53					
Caucasian	3,292	95.81					
Born in UK	3,292	91.28					
Marital/partnership status	3,292						
1. Single		10.15					
2. Married		46.05					
3. Common-law/living with partner/living as married		43.17					
4. Divorced		0.27					
5. Widowed		0.00					
6. Separated		0.00					
**Pregnancy associated demographics**							
Gestational weeks at enrollment	3,292		24.71	9.26	25.43	2.14	41.71
1. Primigravida	3,292	48.06					
2. Multigravida	3,292	51.64					
Number of live born children	1,708		1.30	1.11	1.00	0	8
Ever experienced miscarriage	1,711	50.61					
**Weekly substance use among consumers**							
Average number of alcoholic beverages per week before pregnancy	2,324		5.17	8.36	2.5	0.005	180
Average number of alcoholic beverages per week post-pregnancy recognition	269		2.21	3.66	1	0.020	24
Average number of tobacco products per week before pregnancy	853		65.07	51.73	70	0.002	500
Average number of tobacco products per week post-pregnancy recognition	282		30.23	26.92	25	0.100	140
Average number of cannabis products per week before pregnancy	197		8.28	11.43	3	0.100	56
Average number of cannabis products per week post-pregnancy recognition	47		5.58	8.37	2	0.125	35
Depression diagnosis prior to pregnancy	3,292	33.90					
Depression (EPDS score) during pregnancy	3,081		13.28	6.17	14	0	30
EPDS score clinically-elevated (% cases ≥ 13 = major depressive disorder) during pregnancy	3,081	57.06					
Anxiety diagnosis prior to pregnancy	3,292	41.49					
Anxiety (T-Score) during pregnancy	3,080		60.34	8.91	61.3	36.3	82.7
PROMIS Anxiety scale score clinically-elevated (% cases ≥ 60) during pregnancy	3,080	58.05					
Pregnancy-related anxiety (PRAQ) during pregnancy	3,067		23.06	5.92	22	10	40
**COVID-19 stressors during pregnancy**							
Financial difficulties	3,158		25.08	27.06	15	0	100
Perceived personal health threat	3,145		45.59	25.93	50	0	100
Perceived threat to baby's life	3,145		52.70	27.12	50	0	100
Not receiving care	2,956		47.69	30.82	50	0	100
Days in self isolation	2,302		72.55	42.12	84	1	365
Loneliness	3,055		70.87	25.54	75	0	100
Impact on relationships	2,945		47.89	22.51	50	0	100
Distress at perceived peak of COVID-19 pandemic	3,093		6.22	2.35	7	0	10
Distress past 7 days	3,093		4.51	2.65	5	0	10

### 3.2 Substance use

Alcohol was the most commonly reported substance (ever use: 82.69%; pre-pregnancy use: 74.33%; use after pregnancy recognition: 8.60%), followed by tobacco (37.48%, 26.34%, and 8.75%, respectively), cannabis (14.91%, 6.17%, and 1.49%), and illicit drugs (5.47%, 2.07%, and 0.12%). Overall, 13.24% of participants reported never using any alcohol or substances, 19.87% reported no substance use prior to pregnancy, and 83.66% reported abstaining from all substances after pregnancy recognition.

Among those reporting substance use, the mean weekly alcohol intake declined from 5.17 drinks (SD 8.36) pre-pregnancy to 2.21 (SD 3.66) post-pregnancy recognition. For tobacco, the mean weekly products decreased from 65.07 (SD 51.73) to 30.23 (SD 26.92). For cannabis, mean weekly products declined from 8.28 (SD 11.43) to 5.58 (SD 8.37) following pregnancy recognition. Reductions in substance use after pregnancy recognition were statistically significant for all substances (all *p* < 0.001), with effect sizes ranging from small (illicit drugs, *r* = 0.12; cannabis, *r* = 0.20) to moderate (tobacco, *r* = 0.43) and very large for alcohol (*r* = 0.71) (Supplementary Table 1).

### 3.3 Mental health

A prior diagnosis of depression was reported by 33.9% of participants. Among those who completed the EPDS, 57.06% scored above 13, indicating clinically significant depressive symptoms during the pandemic. A history of anxiety disorder was reported by 41.49%, and 58.05% scored 60 or higher on the PROMIS Anxiety Short Form, indicating moderate-to-severe anxiety symptoms during the pandemic. The mean pregnancy-related anxiety (PRAQ) score was 23.06 (SD 5.92).

### 3.4 COVID-19 stressors

With regard to COVID-19 stressors, participants reported a mean score of 25.08 (SD 27.06, scale 0–100) for financial difficulties. Perceived personal health threat averaged 45.59 (SD 25.93), while perceived threat to the baby's life was slightly higher at 52.70 (SD 27.12). The mean score for concerns about not receiving adequate antenatal care was 47.69 (SD 30.82). Psychological distress at the perceived peak of the pandemic averaged 6.22 (SD 2.35, scale 0–10).

### 3.5 Association between consumption and mental health symptoms

Spearman's rank correlation analyses showed that tobacco use after pregnancy recognition was weakly but significantly associated with higher depressive symptoms (ρ = 0.116, *p* < 0.001), anxiety (ρ = 0.093, *p* < 0.001), and pregnancy-related anxiety (ρ = 0.070, *p* < 0.001), whereas cannabis use after pregnancy recognition was weakly associated with depressive symptoms (ρ = 0.054, *p* < 0.01) and anxiety (ρ = 0.060, *p* < 0.001) (Supplementary Figure S1). Additionally, linear regression analyses were conducted to examine the association between prenatal alcohol, tobacco, and cannabis use and symptoms of depression and anxiety (Table 2). We found that prenatal tobacco use was significantly associated with depression [B = 0.212, SE = 0.063, 95% CI (0.088–0.336), *p* = 0.001], with a small standardized effect size (β = 0.113) as shown in Table 2.

**Table 2:** Linear regression models showing mental health symptoms associated with alcohol/substance use.

**Mental health symptoms**	***N***	**Unstandardized B**	**SE**	**Standardized ß**	***t***	***p***	**95% CI for B**	
							**Lower**	**Upper**
**Alcohol**	3,292							
^†^Depression		0.012	0.007	0.061	1.815	0.070	−0.001	0.025
Anxiety		−0.009	0.006	−0.050	−1.468	0.142	−0.022	0.003
Constant		0.210	0.075		2.796	0.005	0.063	0.358
**Tobacco**	3,292							
**Depression**		**0.212**	**0.063**	**0.113**	**3.361**	**0.001**	**0.088**	**0.336**
Anxiety		−0.055	0.060	−0.031	−0.914	0.361	−0.174	0.063
Constant		0.899	0.718		1.253	0.210	−0.508	2.307
**Cannabis**	3,292							
Depression		0.001	0.007	0.004	0.114	0.910	−0.013	0.014
Anxiety		0.004	0.007	0.019	0.571	0.568	−0.009	0.016
Constant		−0.001	0.077		−0.010	0.992	−0.152	0.151

Analyses were restricted to substance use post-pregnancy recognition. Bold values indicate results that survived false discovery rate correction using the Benjamini-Hochberg step-up procedure, based on comparison of ordered *p*-values to BH significance thresholds. ^†^Associations that remained significant after controlling for covariates (demographics, mental health before pregnancy, use of the substances in the year prior to pregnancy).

### 3.6 Association between consumption and COVID stressors

With respect to COVID-19-related stressors, prenatal tobacco use showed small but statistically significant positive correlations with financial difficulties (ρ = 0.147, *p* < 0.001), perceived threat to the baby (ρ = 0.063, *p* < 0.001), perceived personal health threat (ρ = 0.040, *p* < 0.05), and not receiving adequate care (ρ = 0.063, *p* < 0.001), and a weak negative correlation with social isolation (ρ = −0.048, *p* < 0.05). Prenatal cannabis use was weakly and positively correlated with financial difficulties (ρ = 0.085, *p* < 0.001). Prenatal alcohol use showed weak negative correlations with perceived threat to the baby (ρ = −0.063, *p* < 0.001) and perceived personal health threat (ρ = −0.064, p <0.001), with no other COVID-19 stressors significantly associated (Supplementary Figure S2). Linear regression showed that alcohol use following pregnancy recognition was not significantly associated with any COVID stressors. However, prenatal cannabis use was associated with greater financial difficulties during the pandemic [B = 0.002, SE = 0.001, 95% CI (0.001–0.004), *p* = 0.002, ß = 0.054] (Table 3). Tobacco use was associated with higher perceived personal health threat [B = 0.021, SE 0.008; 95% CI (0.006–0.036); *p* = 0.006; β = 0.049] and greater concerns about inadequate care [B = 0.019, SE = 0.008; 95% CI (0.003–0.035); *p* = 0.018; β = 0.051].

**Table 3:** Linear regression models showing COVID-19 factors associated with alcohol/substance use.

**COVID-19 factors**	***N***	**Unstandardized B**	**SE**	**Standardized ß**	***t***	***p***	**95% CI for B**	
							**Lower**	**Upper**
**Alcohol**	3,292							
Financial difficulties	3,158	0.000	0.001	0.000	−0.009	0.993	−0.002	0.002
Constant		0.180	0.029		6.156	<.001	0.123	0.238
Perceived threat to baby	3,145	0.000	0.001	0.000	0.021	0.983	−0.002	0.002
Constant		0.179	0.044		4.118	<.001	0.094	0.265
Perceived personal health threat	3,145	−0.001	0.001	−0.014	−0.761	0.447	−0.002	0.001
Constant		0.212	0.047		4.506	<.001	0.120	0.304
Not receiving care	2,956	0.000	0.001	0.000	0.001	0.999	−0.002	0.002
Social isolation		−0.001	0.001	−0.027	−1.272	0.204	−0.002	0.000
Constant		0.237	0.066		3.609	<.001	0.108	0.366
**Tobacco**	3,292							
Financial difficulties	3,158	0.000	0.001	0.000	−0.009	0.993	−0.002	0.002
Constant		0.180	0.029		6.156	<.001	0.123	0.238
Perceived threat to baby	3,145	0.005	0.008	0.011	0.600	0.548	−0.011	0.020
Constant		2.372	0.417		5.691	<.001	1.555	3.189
**Perceived personal health threat**	**3,145**	**0.021**	**0.008**	**0.049**	**2.752**	**0.006**	**0.006**	**0.036**
Constant		1.489	0.450		3.309	0.001	0.607	2.371
**Not receiving care**	**2,956**	**0.019**	**0.008**	**0.051**	**2.359**	**0.018**	**0.003**	**0.035**
Social isolation		−0.009	0.006	−0.032	−1.506	0.132	−0.021	0.003
Constant		2.326	0.628		3.703	<.001	1.094	3.558
**Cannabis**	3,292							
**Financial difficulties**	**3,158**	**0.002**	**0.001**	**0.054**	**3.031**	**0.002**	**0.001**	**0.004**
Constant		0.023	0.030		0.775	0.438	−0.036	0.082
Perceived threat to baby	3,145	0.000	0.001	−0.008	−0.427	0.669	−0.002	0.001
Constant		0.102	0.045		2.276	0.023	0.014	0.190
Perceived personal health threat	3,145	−0.001	0.001	−0.014	−0.761	0.447	−0.002	0.001
Constant		0.212	0.047		4.506	<.001	0.120	0.304
Not receiving care	2,956	−0.001	0.001	−0.016	−0.731	0.465	−0.002	0.001
Social isolation		0.000	0.001	0.001	0.046	0.963	−0.001	0.001
^†^Constant		0.113	0.068		1.679	0.093	−0.019	0.246

Analyses were restricted to substance use post-pregnancy recognition. Bold values indicate results that survived false discovery rate correction using the Benjamini-Hochberg step-up procedure, based on comparison of ordered *p*-values to BH significance thresholds. ^†^Associations that remained significant after controlling for covariates (demographic, mental health before and during pregnancy, as well as other COVID-19 stressors).

### 3.7 Association between substance co-use with mental health and COVID stressors

**Figure 1:**
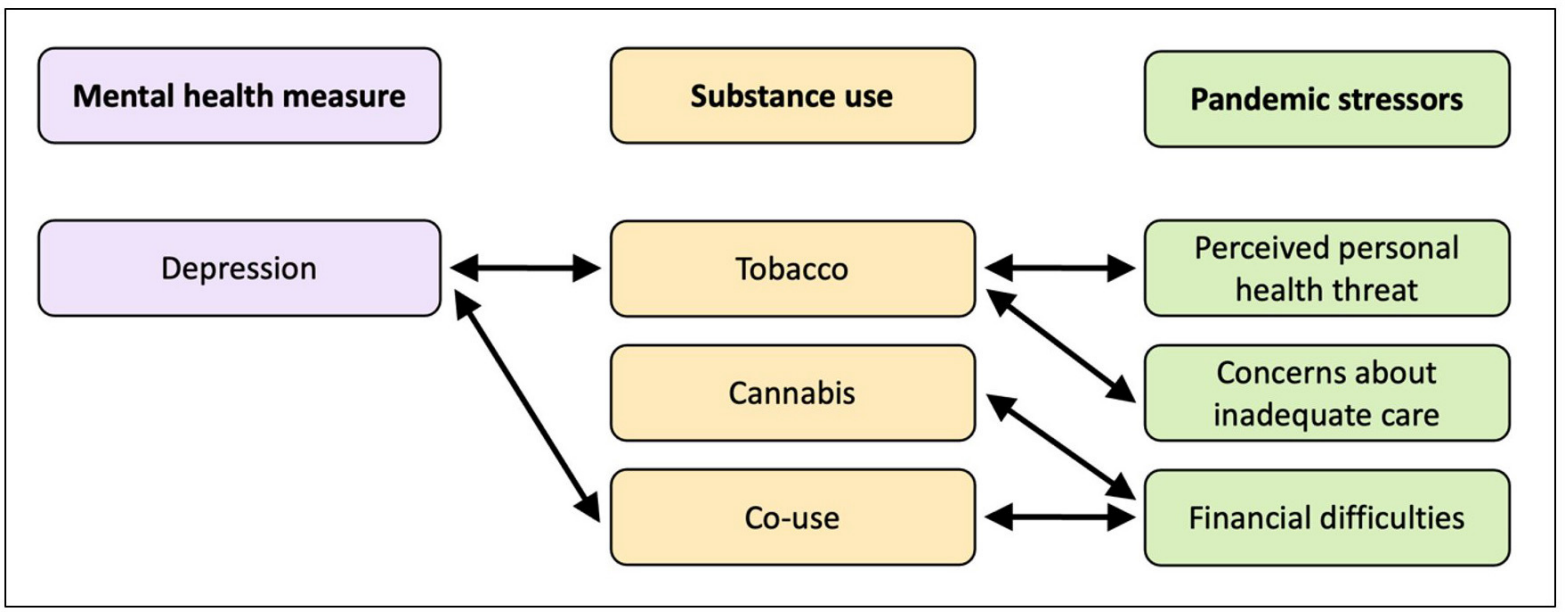
Conceptual framework illustrating hypothesized relationships among pandemic stressors, prenatal maternal mental health symptoms, and substance use following pregnancy recognition. Bidirectional arrows summarize statistical associations observed; no casual direction is implied.

Co-use of substances (simultaneous use of two or more of alcohol, tobacco, and cannabis post-pregnancy recognition) was associated with depressive symptoms [B = 0.002, SE = 0.001, 95% CI (0.000–0.003), *p* = 0.027, ß = 0.075] (Table 4), as well as with financial difficulties during the pandemic [B = 0.000, SE = 0.000, 95% CI (0.000–0.001), *p* < .001, ß = 0.067] (Table 5). A conceptual summary of all the observed associations in the study is presented in Figure 1.

**Table 4:** Linear regression models showing mental health symptoms associated with alcohol/substance co-use.

**Mental health symptoms**	***N***	**Unstandardized B**	**SE**	**Standardized ß**	***t***	***p***	**95% CI for B**	
							**Lower**	**Upper**
**Alcohol/substance co-use**	3,080							
**Depression**	**3,080**	**0.002**	**0.001**	**0.075**	**2.210**	**0.027**	**0.000**	**0.003**
Anxiety	3,080	−0.001	0.001	−0.029	−0.848	0.396	−0.002	0.001
Constant		0.011	0.009	0.000	1.252	0.211	−0.006	0.029

Analyses were restricted to substance use post-pregnancy recognition. Bold values indicate results that survived false discovery rate correction using the Benjamini-Hochberg step-up procedure, based on comparison of ordered *p*-values to BH significance thresholds. There were no significant findings after controlling for covariates (demographics and mental health before pregnancy).

**Table 5:** Linear regression models showing COVID-19 factors associated with alcohol/substance co-use.

**COVID-19 factors**	***N***	**Unstandardized B**	**SE**	**Standardized ß**	***t***	***p***	**95% CI for B**	
							**Lower**	**Upper**
**Alcohol/substance co-use**	3,158							
**Financial difficulties**	**3,158**	**0.000**	**0.000**	**0.067**	**3.795**	**<0.001**	**0.000**	**0.001**
Constant		0.012	0.003	0.000	3.499	<.001	0.005	0.019
Perceived threat to baby	3,145	0.000	0.000	0.019	1.053	0.293	0.000	0.000
Constant		0.016	0.006	0.000	2.848	0.004	0.005	0.027
Perceived personal health threat	3,145	0.000	0.000	−0.001	−0.056	0.955	0.000	0.000
Constant		0.022	0.005	0.000	4.138	<.001	0.011	0.032
Not receiving care	2,956	0.000	0.000	0.033	1.771	0.077	0.000	0.000
Constant		0.014	0.005	0.000	2.832	0.005	0.004	0.023
Social isolation	2,302	0.000	0.000	−0.032	−1.540	0.124	0.000	0.000
Constant		0.029	0.006	0.000	4.847	<.001	0.017	0.040

Analyses were restricted to substance use post-pregnancy recognition. Bold values indicate results that survived false discovery rate correction using the Benjamini-Hochberg step-up procedure, based on comparison of ordered *p*-values to BH significance thresholds. There were no significant findings after controlling for covariates (demographic, mental health before and during pregnancy, as well as other COVID-19 stressors).

### 3.8 Qualitative report

A thematic analysis of 380 responses to the free-text question, “If you would like to tell us anything else about your use of alcohol, tobacco, cannabis, or illicit drugs while pregnant, please do so here,” identified three main themes.

#### 3.8.1 Continued substance use until pregnancy awareness

Many participants reported continuing alcohol, tobacco, or cannabis use until they became aware of pregnancy. In several cases, delayed pregnancy recognition resulted in continued substance use into early pregnancy. Alcohol was most frequently mentioned, although similar patterns were reported for tobacco and cannabis. Participants often described this use as a continuation of their usual consumption prior to pregnancy awareness, followed by cessation once pregnancy was confirmed.

“*I didn't know I was pregnant till 16/17 weeks gone, hence the use of alcohol, cigarettes, and cannabis. I stopped immediately after finding out.”*

“*I did not know I was pregnant until over 8 weeks into my pregnancy. So, I drank alcohol over the Xmas/new year period which was more frequent than I would normally drink.”*

#### 3.8.2 Vaping as a harm-reduction strategy

Several participants described using vaping, often with reduced nicotine concentration, and/or nicotine replacement products as a strategy to decrease cigarette smoking during pregnancy. For some, vaping was adopted after pregnancy recognition as a harm-reduction approach, while others reported having switched from cigarettes to vaping prior to pregnancy and continuing this practice during pregnancy.

“*I smoke a vape with 3 mg of nicotine, which was reduced from 12 mg.”*

“*I have been clean and sober for 10 years. Have transitioned to a mix of nicotine gum and occasional vape while pregnant.”*

“*Regular vaper for 3 years prior to pregnancy after switching from 20 plus cigarettes a day. Occasional cigarettes at work prior to pregnancy, none since. But continuing to vape.”*

#### 3.8.3 Influence of midwifery guidance

Midwives were frequently cited as shaping decisions about substance use. Participants reported receiving advice suggesting that vaping, compared with traditional cigarettes, was unlikely to cause significant harm, particularly when weighed against the potential effects of stress or anxiety.

“*Since finding out I was pregnant I now occasionally have a few drags of a low strength vape a few times per day. I was told by a midwife that this was fine and shouldn't cause any harm to my baby.”*

“*I use a vape with a low nicotine content and was told by my midwife it was safe to continue doing so whilst pregnant because vapes are a lot less harmful than cigarettes. She has monitored my CO throughout pregnancy and this has been the equivalent of a non-smokers.”*

“*Advised to not feel too guilty about smoking as I have used it as a coping mechanism for BPD* [=Borderline Personality Disorder] *and severe anxiety for 10*+ *years. Midwife and therapist said the level of stress could be just as harmful.”*

Together, these themes underscore the heterogeneity of substance use around pregnancy recognition and the centrality of informational and clinical support in participants' narratives.

## 4 Discussion

### 4.1 Summary of main findings

This study examined substance use among pregnant individuals in the UK during the COVID-19 pandemic, focusing on associations with maternal mental health and pandemic-related stressors. Alcohol, tobacco, and cannabis use declined markedly once pregnancy was recognized, yet a concerning subset continued use, often in the context of psychological distress and pandemic challenges. Notably, bivariate correlations between mental health, pandemic stressors, and substance use were generally weak, highlighting the value of modeling approaches for capturing the nature and strength of associations not apparent in simple correlations. Linear regression models showed that tobacco use was associated with depressive symptoms and pandemic stressors, cannabis use with financial strain, and co-use with both depression and financial strain, however, many associations lost statistical significance after covariate adjustment. Qualitative findings highlight early substance use in pregnancy, the use of vaping as a perceived safer-use strategy, and the influence of midwifery advice on decisions about substance use. Together, these results highlight the complex interplay of mental health, stress, and prenatal substance use during a period of societal disruption.

The prevalence of alcohol abstinence in our sample (91.4%) is comparable to a recent UK study of 836 pregnant individuals reporting 91% abstinence (32). This contrasts with prior estimates placing the UK among the highest globally for drinking during pregnancy (2, 22, 33). The consistency of our findings with more recent data suggests a possible decline in alcohol use during pregnancy in the UK, though exposure in early stages of pregnancy remains common.

### 4.2 Maternal depression and pandemic stressors were linked to tobacco use and co-use

Prenatal maternal mental health is widely recognized as a factor influencing substance use (34), and the COVID-19 pandemic further aggravated these challenges (17, 35). Indeed, the UK showed the highest levels of prenatal depression during the pandemic compared with five other nations (20). In our cohort, maternal depression was associated with tobacco use and substance co-use in unadjusted analyses; however, these associations attenuated and did not remain statistically significant after covariate adjustment. Nevertheless, the observed clustering of depressive symptoms with substance co-use is noteworthy, given the established link between co-use and more severe adverse birth outcomes compared with single-substance exposure (36). Nicotine's short-term mood-elevating effects via dopaminergic pathways may partly explain the tobacco- depression association (37), while co-use may reflect escalating coping patterns when one substance alone does not provide sufficient relief.

We also found that tobacco use was associated with pandemic stressors, specifically perceived threat to life and concerns about inadequate care. These associations may reflect gaps in healthcare provision: restricted access during COVID-19 could have reduced opportunities for tobacco cessation counseling, leaving pregnant individuals without guidance on risks or strategies to quit (38).

### 4.3 Financial strain was linked to cannabis use following pregnancy recognition

In our cohort, financial strain during the pandemic was associated with cannabis use post-pregnancy recognition. One possible explanation is that cannabis may have been perceived as a coping strategy under financial strain, potentially offering longer-lasting effects compared with alcohol or tobacco. However, this interpretation remains speculative and warrants further investigation. Prior work suggests that substance choice is shaped not only by absolute cost but also by perceived utility and effect (39). Our findings are consistent with broader literature linking downward socioeconomic mobility and financial difficulties with persistent cannabis use (40). Similar associations were observed in the Canadian Pregnancy During the Pandemic cohort (41), while neither Canadian nor UK data showed associations between alcohol use and mental health or COVID-19 stressors. Together, these findings suggest a cross-national pattern linking prenatal cannabis use to financial stress.

Moreover, previous research, in the United States, has identified motivations for prenatal cannabis use including managing pregnancy-related nausea, pain or sleep (42, 43). In our qualitative sample, only one participant reported using cannabis specifically to alleviate morning sickness and stimulate appetite, suggesting that this motivation was rare in our UK cohort. This difference may reflect variations in legal status, healthcare guidance, cultural norms, or perceptions of safety, which could influence both the decision to use cannabis and willingness to report such use during pregnancy.

### 4.4 Qualitative insights underline the importance of early pregnancy awareness and guidance in maternal health

The qualitative findings from this study revealed important insights into prenatal substance use, particularly highlighting the prevalence of unintentional exposure due to delayed pregnancy recognition. Consistent with the quantitative findings showing substantial reductions in alcohol, tobacco, and cannabis use following pregnancy recognition, the qualitative accounts suggest that continued substance use in early pregnancy may, in some cases, reflect delayed awareness of pregnancy rather than intentional continuation. Participants reported using substances until they became aware of pregnancy, typically between the 4th and 8th gestational week. This timing is concerning, as it coincides with early organogenesis, a critical phase when the developing fetus is highly susceptible to environmental insults (44). There is no known safe level of alcohol during pregnancy, and even early exposure has been linked to neurodevelopmental deficits, miscarriage, and FASD (45). Similarly, tobacco exposure in early pregnancy is associated with an increased risk for adverse outcomes such as placental damage and stillbirth (46). The prevalence of unintentional exposure underscores the potential importance of pre-conception health education, as pregnancy recognition often occurs after critical windows of fetal development have begun and interventions initiated only after recognition may not fully prevent early harm. The reduction in alcohol use following pregnancy recognition was notably larger than that observed for tobacco, cannabis, or illicit drugs (*r* = 0.71 vs. *r* = 0.43, 0.20, and 0.12, respectively), indicating that alcohol-related behavior shifted most markedly once pregnancy was confirmed. This differential pattern may partly reflect clearer and more strongly internalized public health messaging around alcohol use in pregnancy in the UK, as well as the fact that alcohol consumption in early pregnancy is often episodic rather than physiologically dependent. In contrast, nicotine and cannabis use may be more closely tied to dependence or coping mechanisms, making cessation more difficult despite pregnancy recognition. However, differences in baseline prevalence and patterns of use across substances may also have influenced the magnitude of observed reductions. This pronounced behavioral shift nonetheless underscores the temporal limitation of change, as exposure may already have occurred during early gestation prior to pregnancy awareness.

Although this study was conducted before the more recent rise in vaping prevalence, participants described using e-cigarettes as a harm-reduction strategy when attempting to reduce or replace cigarette smoking during pregnancy. These accounts suggest that vaping was often adopted in the context of difficulty quitting nicotine altogether, rather than as a preferred or recreational behavior. This is particularly relevant given the quantitative findings linking prenatal tobacco use with depressive symptoms and pandemic-related stressors, indicating that challenges in cessation may be amplified during periods of psychological strain. While E-cigarettes are marketed as less harmful than traditional cigarettes, evidence suggests that aerosols still contain toxicants, heavy metals (cadmium, lead), and harmful flavoring agents such as diacetyl, which is linked to lung disease (47, 48). E-cigarette use has also been associated with adverse birth outcomes, including low birth weight and preterm birth (47).

Taken together, these findings highlight a tension between harm-reduction approaches adopted by pregnant individuals and the evolving evidence base regarding the safety of vaping in pregnancy. For some individuals, harm-reduction strategies may represent transitional steps toward cessation rather than endpoints. While vaping may be perceived as a means of reducing risk when cessation is difficult, particularly under conditions of stress or poor mental health, it is important to emphasize that complete nicotine cessation remains the safest option for maternal and fetal health during pregnancy.

Our findings also highlight the influential role of midwives in shaping substance use decisions during pregnancy. Participants described receiving mixed or reassuring messages about low-level nicotine use via vaping, suggesting that the way guidance is communicated can shape perceptions of risk. While the UK Chief Medical Officers revised their guidance in 2016 to recommend abstinence from substances during pregnancy or when planning pregnancy (49), subsequent research has highlighted inconsistencies in how such guidance is understood and communicated. A qualitative study conducted in the UK in 2022 found that many midwives were uncertain about the updated alcohol guidelines, pointing to challenges in translating national recommendations into consistent clinical messaging (50).

Importantly, these challenges must be understood within the broader context of maternity care delivery. UK midwives are working under substantial and well-documented system pressures, including chronic understaffing, high workloads, and widespread burnout, which can limit the time and resources available for detailed substance-use screening, counseling, and follow-up (51). In this context, variability in guidance may reflect structural constraints within maternity care, including limited opportunities for training and the practical challenges of delivering detailed counseling within time-pressured clinical settings.

Qualitative studies from Australia have explored midwives' experiences of caring for pregnant women who use substances, highlighting empathetic and non-judgmental approaches, with midwives recognizing substance use as shaped by social, environmental, and life-course circumstances rather than individual choice alone (52). These studies also describe the complexity of providing care in this context and emphasize the need for clearer referral pathways and targeted training to support effective screening and intervention (53). In particular, training that supports clear, consistent, and non-stigmatizing communication around substance use, including products such as e-cigarettes and vaping, may help facilitate open disclosure and trust-based communication about substance use within antenatal care. Future studies should consider collecting more comprehensive data on midwifery advice and support related to substance use, including vaping, to enable more detailed examination of how guidance interacts with mental health and substance use during pregnancy.

### 4.5 Public health implications

Our findings emphasize the need for universal pre-conception education on alcohol, tobacco, and cannabis, together with routine screening for both substance use and mental health disorders in antenatal care. Substance use during pregnancy should not be regarded merely as a lifestyle choice, but is frequently rooted in psychological distress and socioeconomic pressures. Effective strategies must therefore address individual behaviors as well as structural determinants, including financial strain and access to healthcare. Pre-conception counseling should emphasize cessation before conception to minimize early fetal exposure. Midwives and other healthcare providers should be equipped to deliver consistent, evidence-based, and personalized counseling to support these decisions. Inconsistent or unclear recommendations undermine trust and ensuring that guidance is credible and coherent is particularly important in the UK, where professional and public confusion persists (49). Ultimately, a holistic approach that integrates mental health support and substance use counseling is essential, both during global health crises and in routine maternal care.

### 4.6 Strengths, limitations, and conclusion

This is the first large-scale study in the UK to investigate prenatal substance use in the context of the COVID-19 pandemic. Strengths include the large sample size, the mixed-methods design, which provided both quantitative associations and qualitative insights and the use of validated mental health scales. Although the Distress Thermometer (DT) was originally developed and validated in oncology settings (54), it has subsequently been applied in perinatal research as a brief screening measure of general psychological distress, including during the COVID-19 pandemic (29, 55). In the present study, the DT was used to capture acute, non-specific distress rather than to provide a diagnostic assessment of depressive or anxiety disorders.

Several limitations should also be considered when interpreting these findings. First, “prenatal use” referred to any use after pregnancy detection and we could not distinguish between exposure limited to the early weeks vs. continued use throughout pregnancy. Second, the higher percentages of participants reporting a prior diagnosis of depression and anxiety indicate that healthier women may be underrepresented, while individuals with pre-existing mental health difficulties may be over-represented in our sample. This enrichment could potentially inflate observed associations between depression, anxiety, and substance use and limits the generalizability of our findings. Moreover, the predominantly Caucasian, UK-born composition of the cohort limits the applicability of our findings to racially and ethnically diverse populations. Patterns of substance use and disclosure may vary across cultural, religious, and social contexts, including norms related to alcohol and drug use, stigma, and concerns about disclosure, which may influence both behavior and reporting (56). Substance use may have been under-reported, including in our cohort, due to concerns that disclosure could prompt child protective services involvement or other legal and social repercussions- such as increased surveillance, referral to social services, or adverse consequences for custody or parental rights (57, 58). These factors should be considered when interpreting the implications of our findings for broader public health policy. Finally, although regression analyses accounted for covariates, many associations did not remain significant after adjustment, highlighting that these links may be sensitive to confounding; underlining the complexity of factors influencing substance use in pregnancy.

In conclusion, while the majority of participants discontinued alcohol, tobacco, and cannabis once they recognized their pregnancy, a concerning minority continued use, often related to late pregnancy detection, depressive symptoms, or socioeconomic stressors such as financial strain and inadequate prenatal care. Integrating routine screening for mental health and substance use into prenatal care, coupled with pre-conception education and consistent counseling from healthcare providers, including midwives, could help reduce prenatal exposures. Public health strategies should also prioritize addressing socioeconomic determinants of health and barriers to care. Such holistic approaches are essential not only in times of crisis, but also as part of routine maternal health care, to safeguard the long-term wellbeing of both mother and child.

## Data Availability

The raw data supporting the conclusions of this article will be made available by the authors, without undue reservation.
