# Supplementary material for: Prenatal substance use during the COVID-19 pandemic in the United Kingdom: associations with depression, anxiety, and pandemic stressors
Source: Front Public Health. 2026 Mar 19;14:1760266. doi: 10.3389/fpubh.2026.1760266 (PMC13044118; doi:10.3389/fpubh.2026.1760266)
Supplement: Supplementary file 1 [file Table_1.docx]

**Supplementary Table 1: Paired comparisons of substance use indices before pregnancy and after pregnancy recognition using Wilcoxon signed-rank tests.**

| **Measure 1** | **Measure 2** | **N** | **W** | **z** | **r** | **p** |
| --- | --- | --- | --- | --- | --- | --- |
| Pre-pregnancy alcohol index | Post-pregnancy recognition alcohol index | 3292 | 2,627,000 | −40.87 | 0.71 | < .001 |
| Pre-pregnancy tobacco index | Post-pregnancy recognition tobacco index | 3292 | 338,206 | −24.62 | 0.43 | < .001 |
| Pre-pregnancy cannabis index | Post-pregnancy recognition cannabis index | 3292 | 17,305 | −11.49 | 0.20 | < .001 |
| Pre-pregnancy  illicit drug index | Post-pregnancy recognition illicit drug index | 3292 | 2,260 | −7.00 | 0.12 | < .001 |

Effect sizes were calculated as r = |z|/√N, where N represents the number of paired observations included in each test. Indices reflect combined frequency and quantity of use, defined as the number of days per week and the number of drinks or substances per day in a typical week before and after pregnancy recognition. p < 0.001 indicates that the observed declines in substance use after pregnancy recognition were statistically significant.
