# Supplementary material for: Prenatal substance use during the COVID-19 pandemic in the United Kingdom: associations with depression, anxiety, and pandemic stressors
Source: Front Public Health. 2026 Mar 19;14:1760266. doi: 10.3389/fpubh.2026.1760266 (PMC13044118; doi:10.3389/fpubh.2026.1760266)
Supplement: Supplementary file 2 [file Data_Sheet_1.docx]

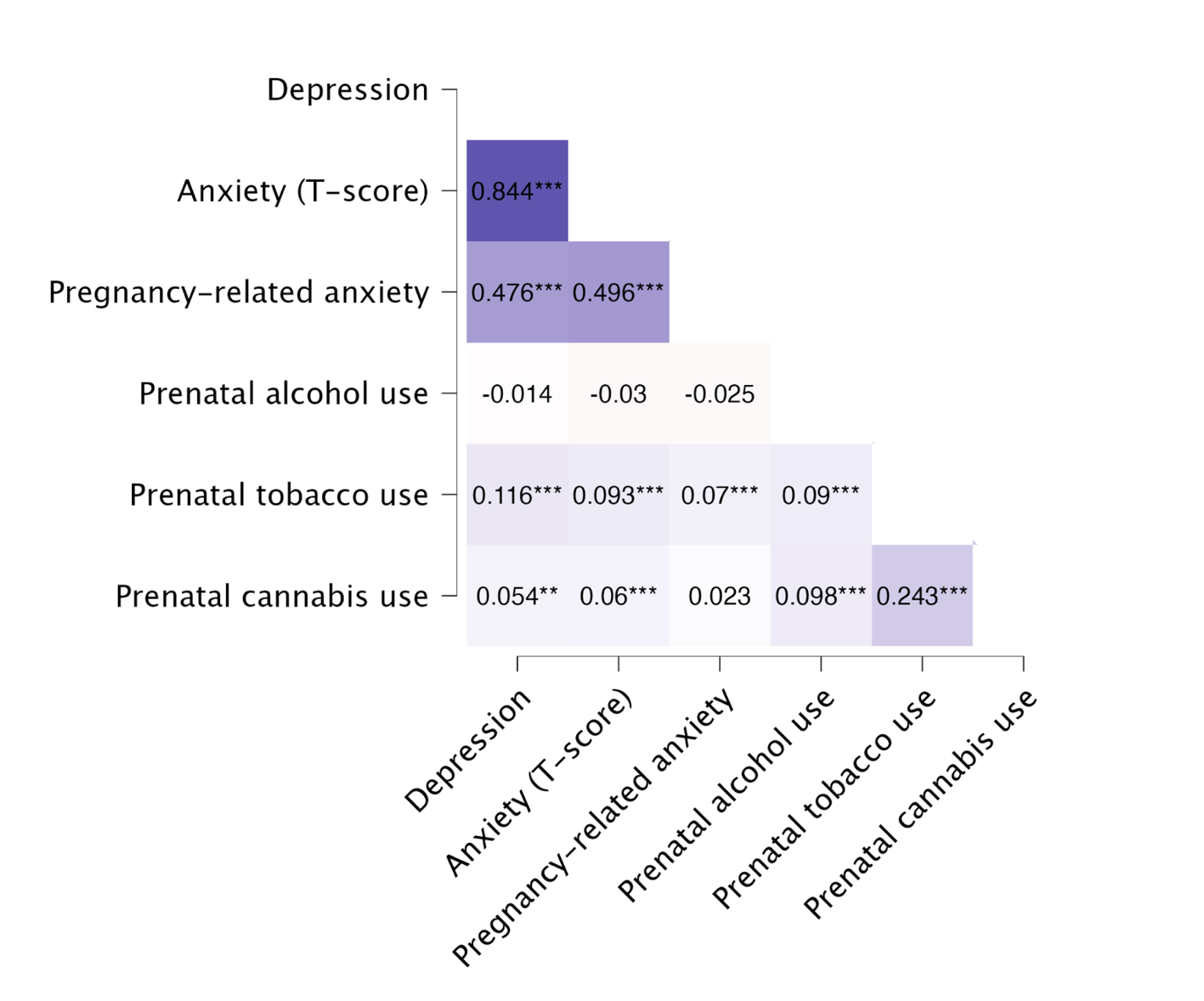


**Supplementary Figure S1: Spearman’s rank correlation matrix of maternal mental health variables and substance use after pregnancy recognition.**

* p < 0.05, ** p < 0.01, *** p < 0.001.


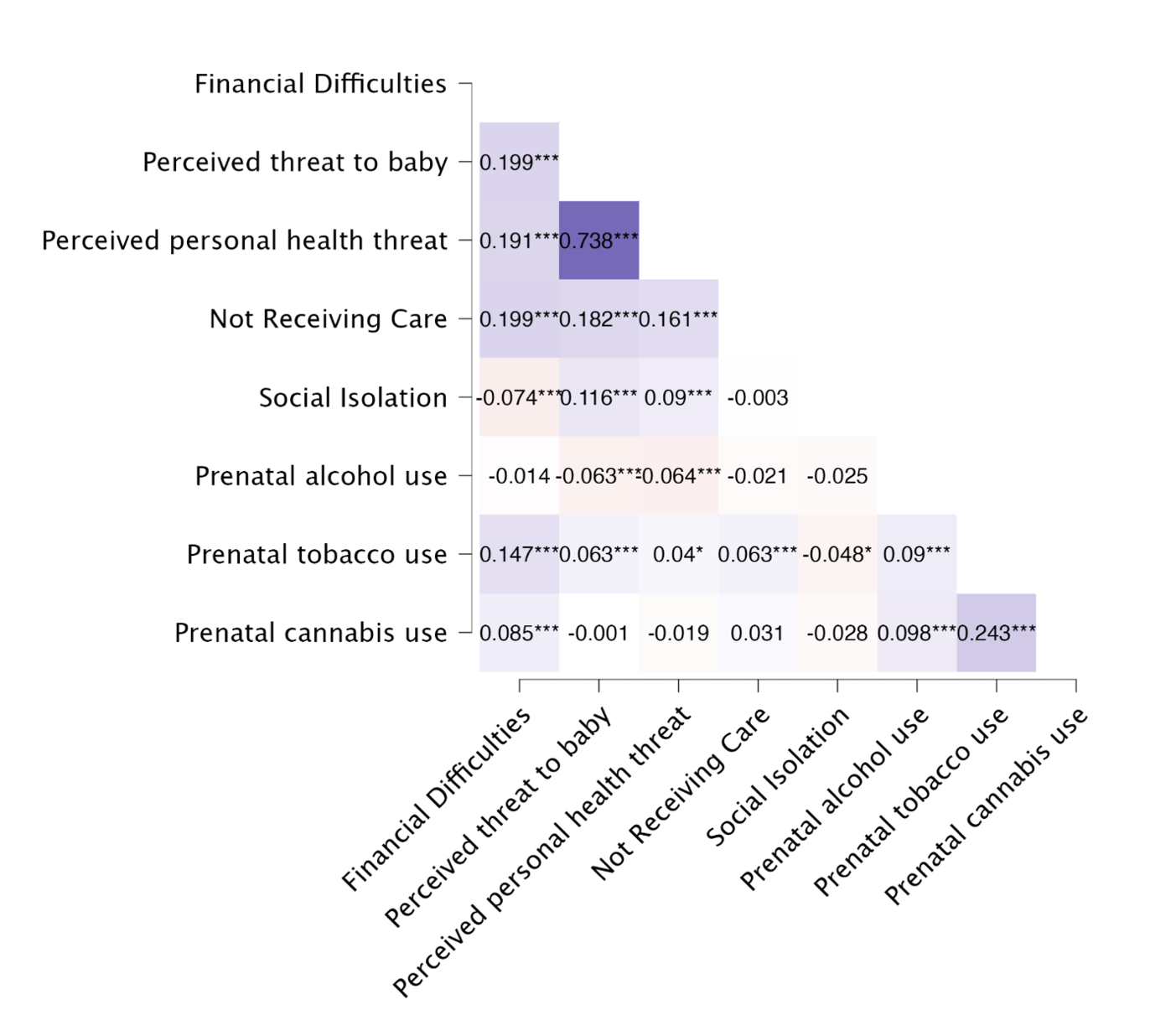


**Supplementary Figure S2: Spearman’s rank correlation matrix of pandemic-related stressors and substance use after pregnancy recognition.**

* p < 0.05, ** p < 0.01, *** p < 0.001.
